# Bioactive Compounds for Fibromyalgia-like Symptoms: A Narrative Review and Future Perspectives

**DOI:** 10.3390/ijerph19074148

**Published:** 2022-03-31

**Authors:** Chwan-Li Shen, Alexis Schuck, Christina Tompkins, Dale M. Dunn, Volker Neugebauer

**Affiliations:** 1 Department of Pathology, Texas Tech University Health Sciences Center, Lubbock, TX 79430, USA; dale.dunn@ttuhsc.edu; 2 Center of Excellence for Integrative Health, Texas Tech University Health Sciences Center, Lubbock, TX 79430, USA; volker.neugebauer@ttuhsc.edu; 3 Center of Excellence for Translational Neuroscience and Therapeutics, Texas Tech University Health Sciences Center, Lubbock, TX 79430, USA; 4 Department of Medical Education, Texas Tech University Health Sciences Center, Lubbock, TX 79430, USA; alexis.schuck@ttuhsc.edu (A.S.); christina.tompkins@ttuhsc.edu (C.T.); 5 Department of Pharmacology & Neuroscience, Texas Tech University Health Sciences Center, Lubbock, TX 79430, USA; 6 Garrison Institute on Aging, Texas Tech University Health Sciences Center, Lubbock, TX 79430, USA

**Keywords:** functional food, fibromyalgia, inflammation, mitochondria, nervous system

## Abstract

Fibromyalgia (FM) is a prevalent, chronic condition without a cure or reliable therapy. The etiopathogenesis of this syndrome is ambiguous, which has heightened the challenge of discovering treatments to minimize patients’ painful symptoms. FM is characterized by diffuse musculoskeletal pain usually accompanied by functional pain syndromes, such as fatigue, sleep disturbances, cognitive difficulties, and mood issues. Currently available treatment options for FM are limited. Recent studies have suggested a potential role for dietary bioactive compounds in FM management. We performed a narrative review to evaluate the existing evidence regarding the dietary bioactive compounds for FM, and we proposed molecular mechanisms on this topic. The inclusion criteria were (i) human, in vivo, or in vitro studies, (ii) studies related to the effect of bioactive compounds on FM-like symptoms, (iii) peer-reviewed literature, and (iv) publications until February 2022 in PubMed and Google Scholar. Exclusion criteria were (i) study designs using CCI, SNI, or SNL models because they are more NP models rather than FM models, and (ii) studies published in a language other than English. Keywords were dietary bioactive compounds, fibromyalgia, cell, animals, humans. Here, we report the effects of commonly consumed bioactive compounds (capsaicin, ginger, curcumin, n-3 PUFA, grape seed extract, naringin, and genistein) on FM-like symptoms in cellular, animal, and human studies. Cellular studies demonstrated that these bioactive compounds reduce pro-inflammatory production and increase antioxidant capacity of neurons or myoblasts that regulate apoptosis/cell survival. Animal studies showed that these regularly consumed bioactive compounds have an effect on FM-like symptoms, as evidenced by decreased pain hypersensitivity and fatigue as well as improved social behaviors. Further studies are warranted to allow meaningful comparison and quantification of the efficacy of these bioactive compounds on FM-like symptoms across studies, in terms of actual changes in antioxidant capacity, pain hypersensitivity, fatigue, and social behaviors. To date, human studies regarding the efficacy of these bioactive compounds on FM-like symptoms are limited and inconclusive. Our review identifies this important knowledge gap and proposes that the development and use of improved preclinical FM models are needed, particularly concerning the usage of female animals to better mimic FM pathophysiology and symptomatology.

## 1. Introduction

Fibromyalgia (FM) is a chronic, functional illness of neurogenic origin [1,2]. Its prevalence has been estimated to afflict approximately five million Americans over the age of eighteen [3]. Published studies have provided estimates of a predominance from <61% to >90% in females [4]. FM has been associated with reduced income and education, and higher rates of divorce and disability status [5,6]. FM is characterized by widespread pain throughout the body for at least three months and pain or tenderness when a pressure stimulus is applied to so-called tender points of the body, which is usually accompanied by symptoms such as fatigue, sleep disruption, cognitive dysfunctions and mood issues [7]. The etiopathogenesis of FM remains unclear, with genetic factors, environmental triggers, and neuromodulation all possibly contributing to the onset and course of this syndrome [8]. One of the more established possible etiologies involves amplification of central and peripheral pain and sensory processing in the ascending (pro-nociceptive) and descending (anti-nociceptive) pathways [1,9]. This deviant nociceptive processing may play a role in FM patients’ hyperexcitable responses to heat, cold, or other painful stimuli [9].

Growing evidence suggests that mitochondrial dysfunction, oxidative stress, and pro-inflammatory cytokines play a role in the progression of central amplification of pain signals in FM [10]. Impaired mitochondrial function leads to a decreased amount of free radical scavenging in cells, allowing elevated lipid peroxidation and mitochondrial DNA (mtDNA) damage to take place [10]. Increased mitochondrial reactive oxygen species (ROS), such as superoxides, hydrogen peroxides, and hydroxyl radicals may be involved in muscle pain and central amplification by decreasing the amount of ATP available in muscle and neural cells [11]. Superoxides were reported to play a significant role in both peripheral and central nociceptive signaling associated with hyperalgesia in rats, which is an increased pain response or pain sensation to a noxious stimulus [12]. In addition, the expression of pro-inflammatory cytokines related to neuroinflammation in FM-associated functional pain syndrome may be dependent upon and elevated by mitochondrial dysfunction and oxidative stress [10,13,14,15,16]. Neurogenic inflammation, resulting from the release of pro-inflammatory neuropeptides from C-fibers, is also prominent in FM and contributes to allodynia, tissue swelling, and dysesthesia [17]. Indeed, FM patients’ blood mononuclear cells (BMCs) have been found to have decreased coenzyme Q10 (CoQ_10_) and mitochondrial DNA levels and increased mitochondrial ROS and serum tumor necrosis factor-alpha (TNF-α) levels [10]. Mitochondrial dysfunction has also been noted in FM patients’ muscles [11]. As such, FM patients have been treated with nonnarcotic anti-algesic agents that focus on mitochondrial biogenesis, antioxidant, and anti-inflammatory effects [18,19,20].

Morphologically, patients with chronic trapezius myalgia (i) had larger type I fibers and a lower capillary:fiber area ratio for type I and type II-A fibers, and (ii) exhibited lower levels of ATP and phosphocreatine in both type I and type II fibers, suggesting an imbalance between the capillary supply and the cross-sectional fiber area of type I and type II-A fibers [21]. Such imbalance might be of significance in the development of muscular fatigue and pain, which are FM-like symptoms [21]. Compared to healthy subjects without FM, patients with FM have significantly greater upper arm muscle contra-activation in the biceps muscle, suggesting a possible link between muscle dysfunction, mood, and pain in FM patients [22]. On the other hand, a recent observational study reported that the morphology and quality of cervical multifidus muscle were not associated with clinical variables in women with FM [23].

Fibromyalgia occurs without apparent tissue damage or inflammation; it is fundamentally different from other pain conditions such as rheumatoid arthritis or neuropathic pain and requires a distinct set of therapeutic approaches. Available treatments for FM include antidepressants such as selective serotonin reuptake inhibitors (SSRIs), norepinephrine reuptake inhibitors (SNRIs), and tricyclic antidepressants (TCAs) [24]. Furthermore, anticonvulsants (such as gabapentin and pregabalin), cognitive behavioral therapy, non-invasive brain stimulation, and lifestyle modifications (i.e., mind-body exercise Tai Chi, stress management, and improved sleep) have all been used with FM-established patients [8,24]. However, these limited and generally unsatisfactory treatment options for FM call for further investigation into underlying mechanisms.

Progress in the development of new and alternative drugs or interventions for the treatment of FM requires a better understanding of its multifaceted pathophysiological pathways and relevant molecular networks [25,26]. Functional foods/bioactive compounds and secondary metabolites may offer a useful alternative in FM management, due to their invaluable role as a source of new molecules that can act through innovative mechanisms of action. The anti-FM effect of functional foods/bioactive compounds may be ascribed to their capability to directly or indirectly interact with FM-associated oxidative stress and inflammation due to their anti-inflammatory and antioxidant properties. However, a review has been lacking on how commonly consumed dietary bioactive compounds affect FM-like symptoms along with possible molecular mechanisms. Thus, we performed a narrative review to evaluate the existing evidence regarding the dietary bioactive compounds for FM, and we proposed potential molecular mechanisms on this topic. The inclusion criteria were (i) human, in vivo. or in vitro studies, (ii) studies related to the effect of bioactive compounds on FM-like symptoms, (iii) peer-reviewed literature, and (iv) studies published until February 2022 in PubMed and Google Scholar. Exclusion criteria were (i) study designs using CCI, SNI, or SNL models because they are more NP models rather than FM models, and (ii) studies published in a language other than English. Thus, in this narrative review, we summarize the preclinical and human studies reported in the literature and discuss possible mechanisms for the inhibitory effects of generally consumed dietary bioactive compounds, namely capsaicin, ginger, curcumin, grape seed extract, n-3 polyunsaturated fatty acids (PUFA), naringin, and genistein/soy, on FM-like symptoms. We also discuss their possible molecular mechanisms, including mitochondrial biogenesis and antioxidant and anti-inflammatory effects.

Due to the complexity and ambiguity surrounding the etiologies and subtypes of FM, no single animal model has been deemed “ideal” thus far [27]. A variety of models mimic signs and symptoms of FM, producing similar widespread and long-lasting hyperalgesia, a hallmark of the syndrome’s clinical presentation [28]. Specifically, the ideal preclinical model for FM should include predominance in female animals, absence of (peripheral) tissue injury, and kay symptoms and co-morbidities present in FM patients, such as fatigue, disrupted sleep, cognitive difficulties, depression, and anxiety [27]. Repeated muscle insults have been used as FM models (see [24]) and include the acid saline-induced pain model, hyperalgesic priming model, fatigue-enhanced muscle pain model, and biogenic amine depletion model. Other models used to study FM-like symptoms are stress models (cold stress, sound stress, and sub-chronic swim test) [27]. Data obtained with a number of these animal models are included in this review due to their ability to mimic some of the clinical symptoms seen in FM.

## 2. Capsaicin

Capsaicin is a phenolic compound found in chili peppers and is responsible for the characteristic burning and irritant effect associated with ingestion [29]. Table 1 lists the effect of capsaicin on FM-like symptoms.

Shang et al. reported pretreatment of capsaicin suppressed lipopolysaccharide-induced inflammation in myoblast cells [30]. In the development of FM, receptor-mediated mechanisms have been well-defined and include activity on transient receptor potential vanilloid type 1 (TRPV1) and protein kinase activity [29], while non-receptor mediated mechanisms have more recently been proposed, such as antioxidant and anti-inflammatory properties [31]. Vos et al. demonstrated capsaicin’s ability to act as an exogenous agonist on the polymodal nociceptor, TRPV1 [32]. When co-expressed with TRPV1b, which is the human splice variant, the TRPV1 channel functions were inhibited by capsaicin in a dose-dependent manner due to the possible heterocomplex formation between TRPV1 and TRPV1b [32]. Another proposed mechanism for the downregulation of the TRPV1 receptor includes the complete depolarization and eventual desensitization of the receptor after repeated application of capsaicin in high concentrations, which eventually leads to reduced inflammation and chronic pain [33].

**Table 1:** Effects of capsaicin on fibromyalgia-like symptoms.

First Author, Year [ref]	Experimental Design and Treatments	Results
*In vitro studies*		
Shang, 2017 [24]	Myoblast cells pre-exposed to capsaicin (50, 100 μM) overnight and then treated with LPS (100 μg/mL)	↓ LPS-induced inflammation
Vos, 2006 [26]	HEK293 cells transiently transfected with full--length TRPV1 and activated by capsaicin (1 µm)	↓ TRPV1 channel function in a dose-dependent manner
*Animal studies*		
Scheich, 2017 [28]	Chronic restraint stress-induced FM model	Compared to control group, RTX group:
	CD1 male mice (12-week-old, *n* = 9–11/group)	↓ mechanical hyperalgesia
		↑ basal noxious heat threshold
	Treatments: Capsaicin analogue RTX (10, 20, 70, 100 μg/kg) s.c. once daily for four consecutive days	↔ anxiety, depression, or peripheral inflammatory changes
*Human studies*		
McCarty, 1994 [31]	Randomized double-blind, vehicle-controlled trial with primary	Compared to control group, capsaicin group:
	FM patients (*n* = 45, 44 women, 1 man)	↑ grip strength at week 2 ↓ tenderness in tender points at week 4
	Treatments: capsaicin (0.025%) cream applied as thin layer to tender points on left or right side of upper body as directed by the tube label for 4 weeks	↔ Visual Analog Scale of pain scores
		↑ minor side effects including transient burning at application site
Casanueva, 2013 [32]	Randomized controlled trial with FM patients	Compared to control group, capsaicin group:
		↑ desensitization to pain with repeated capsaicin application
	(*n* = 130, 126 women, 4 men)	↓ Fibromyalgia Impact Questionnaire
		↓ Fatigue Severity Scale scores
	Treatments: capsaicin (0.075%) cream applied topically over 18 tender points 3x/day for 6 weeks	↓ myalgia score
		↓ pressure pain threshold
		↓ role limitations due to emotional problems
		↓ Visual Analogue Scale of depression
Chrubasik, 2010 [33]	Randomized double-blind, placebo-controlled trial with chronic soft tissue/back pain patients (*n* = 281, 174 women, 107 men) Treatments: capsaicin (0.05%) cream applied as a thin layer to painful area 3x/day for 3 weeks	Compared to control group, capsaicin group:
		↓ pain sum scores
		↑ minor side effects including local warmth and pruritis

Abbreviations: FM, fibromyalgia; LPS, lipopolysaccharide; RTX, resiniferatoxin (capsaicin analogue); s.c., subcutaneous; TRPV1, transient receptor potential vanilloid-1; ↑ increase; ↓ decrease; ↔ no difference.

An in vivo intermittent cold stress model for FM-like symptoms was performed where capsaicin-sensitive neurons were desensitized by the capsaicin analog resiniferatoxin (RTX) [34]. RTX exhibits the effects of desensitization on chronic restraint stress-induced responses, as shown by decreased mechanical hyperalgesia, decreased heat pain tolerance, and no evidence of accompanying anxiety, depression, or peripheral inflammatory changes in male mice with FM [34]. Mental health issues are common comorbidities with FM, so these findings are important when considering the effectiveness of capsaicin for FM treatment in humans. Reyes-Mendez et al. demonstrated that low doses of capsaicin produced antidepressant-like effects and synergism with amitriptyline, which is a common medication prescribed to FM patients [35].

Various human studies have also been conducted over the years to provide valuable information about not only the effect of topical capsaicin as a potential therapy for FM-like symptoms but also the adverse effects that must be considered in humans, such as the burning sensation upon application. For example, McCarty et al. revealed a decrease in tenderness at tender points and an increase in grip strength in patients with primary FM who applied topical capsaicin cream to tender points on their body as directed [36]. Serious adverse effects were not reported apart from a transient burning sensation at the application site, which usually decreased with repeated applications [36]. Application of topical capsaicin cream was shown to elicit short-term improvements in patients severely affected by FM [37]. Specifically, patients treated with capsaicin noted improvements in pressure pain thresholds, fatigue severity, and myalgic symptoms compared to patients in the control group continuing their normal treatment regimen [37]. The results from this study by Casanueva et al. demonstrated that repeated application of capsaicin cream for six weeks showed a desensitization to pain as well as a decrease in symptoms of depression [38]. Capsaicin cream was also found to reduce the pain sum score in patients suffering from chronic soft tissue or back pain, and the cream was generally well tolerated with local warmth and pruritis being the only adverse effects reported [39,40].

## 3. Ginger

Ginger is a widely used spice containing phenolic compounds such as gingerols and shogaols, which are largely responsible for various health benefits [41]. Other chemical constituents of ginger include terpenes, polysaccharides, lipids, organic acids, and raw fibers [41]. Several in vitro and animal studies demonstrate the various anti-nociceptive properties of ginger, such as anti-inflammatory, anti-oxidative stress, and neuroprotective effects (Table 2).

In vitro studies have examined the antioxidant effects of ginger that potentially relate to FM-like symptoms. Ha et al. reported that incubation with 6-shogoal, a bioactive component of ginger, resulted in a significant decrease in the expression of inducible nitric oxide synthase (iNOS) and therefore a diminished release of nitric oxide (NO) [42]. Hosseinzadeh et al. further reported that ginger extract not only increased anti-oxidant gene expression in human chondrocyte cells but also reduced IL-1β-induced elevation of reactive oxygen species, decreased lipid peroxidation, decreased the Bax/Bcl ratio, and decreased the caspase-3 activity leading to decreased apoptosis [43].

An intermittent cold stress (ICS) model was used on female mice to specifically study the effects of ginger rhizome on FM-like symptoms [44]. Montserrat-de la Paz et al. reported there was a significant decrease in mechanical and thermal allodynia as well as a significant decrease in mechanical hyperalgesia with ginger-supplemented ICS animals [44]. In the same study, authors further demonstrated ginger suppressed pro-inflammatory mediators, such as NO, prostaglandin E_2_ (PGE_2_), Thromboxane B_2_ (TXB_2_), and IL-1β in LPS-induced macrophages [44]. These results indicated an improvement in behavioral changes related to cognitive disturbances associated with pain, anxiety, and depression in the ginger group [44]. Similar mental health issues are common co-morbidities of FM in humans, so this component of the study provides valuable insight into possible applications of ginger in clinical trials.

With a complete Freud’s adjuvant FM-like symptom model, Fajrin et al. reported that administration of red ginger oil decreased FM-like chronic hyperalgesia and increased the thermal response threshold in animals [45]. A chlorpyrifos model was utilized by Abolaji et al. to induce oxidative damage and inflammation in the brain, ovary, and uterus of female rats in another in vivo study. Abolaji et al. reported that 6-gingerol-rich fraction (6-GRF) administration resulted in (i) a decrease in hydrogen peroxide and malondialdehyde (MDA) levels, and (ii) an increase in catalase, superoxide dismutase, and glutathione peroxidase levels, suggesting 6-GRF’s protective effect against oxidative damage [46]. Such anti-inflammatory properties of 6-GRF were shown via decreased levels of NO, myeloperoxidase (MPO), and tumor necrosis factor-α (TNF-α), leading to decreased apoptosis [46]. The dextran sodium sulfate-induced chronic colitis model is useful due to the common comorbidity of irritable bowel syndrome with FM in humans. Zhang et al. reported that administration of ginger-derived nanoparticles led to an accelerated rate of healing in wounded intestinal epithelial monolayers in animals with chronic colitis. Authors also showed there was an increase in the production of anti-inflammatory cytokines, IL-10 and IL-22, as well as a decrease in the production of pro-inflammatory cytokine, MPO, in animals with colitis [47]. Furthermore, ginger was reported to work synergistically with paracetamol, a common pain-killer, in reducing inflammation and FM-like symptoms in an experimental model of FM [44].

## 4. Curcumin

Curcumin is the primary active component of turmeric, which is one of the main ingredients in curry powder. Curcumin has been used for centuries in Indian and Chinese medicine due to its ability to impact a diverse range of molecular targets. Many studies using mice and rats have demonstrated that this bioactive compound has anti-inflammatory and antioxidant potential by suppressing numerous cell-signaling pathways, including nuclear factor kappa-light-chain-enhancer of activated B cells (NF-κB), signal transducer and activator of transcription 3 (STAT3), nuclear factor-erythroid factor 2-related factor 2 (Nrf2), ROS, cyclooxygenase-2 (COX-2), superoxide leukocyte recruitment, and oxidative stress [48]. 

Table 3 lists the effects of curcumin on FM-like symptoms in animal studies. Yang et al. performed an animal study using the dextran sodium sulfate-induced ulcerative colitis model to evaluate the effect of curcumin on animals [49]. Due to the sustained high concentration of curcumin in the gastrointestinal tract, this model offers valuable insight regarding the possible application to FM-like symptoms treatment in humans [49]. Yang demonstrated that visceral hyperalgesia was reduced in a curcumin dose-dependent manner, with no significant effect detectable using a lower curcumin dose [49]. Furthermore, this experiment by Yang et al. showed a decrease in TRPV1 expression in the dorsal root ganglia as well as a decrease in TRPV1 protein level in the inflamed colon [49]. Two experimental FM-like symptoms models on animals were conducted by Zhi et al. to study the effect of curcumin on gastrointestinal nociception. The first experiment implemented a colorectal distension-induced model, which showed that curcumin decreased visceral hyperalgesia due to a decreased viscero-motor response to colorectal distension in rats [50]. The second experiment used ex vivo mouse jejunum preparations to show a decrease in TRPV1 activation and hypersensitivity of jejunal afferent neurons [50]. Interestingly, curcumin attenuated the capsaicin-induced rise of intracellular calcium and inward currents in mouse and rat dorsal root ganglia neurons [50]. Such a downregulation of the TRPV1 receptor due to curcumin can be related to one of the proposed mechanisms of capsaicin in which it initially activates the TRPV1 receptor, but will eventually cause desensitization of the receptor with repeated applications and thus decrease intracellular calcium levels [50].

The antioxidant and anti-inflammatory properties of curcumin were shown in another animal study where KO_2_ was used as a superoxide anion donor and curcumin was administered subcutaneously [51]. The results of this study by Fattori et al. indicated diminished mechanical and thermal hyperalgesia, which were similar to the results from the aforementioned studies of curcumin. Additionally, superoxide anion-induced leukocyte recruitment in the peritoneal cavity of animals was decreased along with the suppression of myeloperoxidase activity, oxidative stress, and pro-inflammatory cytokines [51]. Intriguingly, Arora et al. demonstrated curcumin works similarly to gabapentin by decreasing levels of excitatory neurotransmitters, such as substance P, in animal models [52].

## 5. Grape Seed Extract

Grape seed extract (GSE), rich in polyphenol groups, is a natural plant derivative often produced as a waste byproduct during the winemaking process. Polyphenols include but are not limited to: proanthocyanidins, procyanidins, anthocyanins, gallic acid, catechin, and epicatechin. GSE high in proanthocyanidin content is formally known as grape seed proanthocyanidin extract (GSPE). The neuroprotective, antioxidant, and anti-inflammatory effects have been well reported in in vitro and animal studies [53]. Table 4 lists the effects of GSE and its bioactive compounds on FM-like symptoms.

Fujishita et al. demonstrated that administration of GSE from Koshu groups decreased H2O2-induced neuronal cell death via upregulating the IL-6, COX-2, and IL-1α in astrocytes with oxidative stress states, suggesting GSE’s neuroprotective effect [53]. The study also found the neuroprotective effect stopped with co-treatment of GSE and anti-IL-6 antibody, suggesting how endogenous IL-6 synthesized from astrocytes plays a neuroprotective role in hippocampal neurons [53]. Narita et al. reported that Koshu grapes (rich in higher polyphenol and procyanidin oligomer) protected mitogen-activated protein kinase (MAPK) phosphorylation, dendritic arborization, and decreased apoptotic activity in glutamate treated hippocampal neurons [54].

In terms of FM-like symptoms using animals, Mun et al. reported oligomeric proanthocyanidin complex (OPC) administration had anti-hyperalgesic effects in an acidic saline animal model that mimics fibromyalgia, due to OPC’s antioxidant and anti-inflammatory properties [48]. In the same animal study, authors found a decreased ASIC3 expression, an ion sensing channel present in central and peripheral nervous systems, in the M1 and M2 brains of hyperalgesic animals [48].

Decreased fatigue was also reported by extending the time to exhaustion (TTE) in a mouse model of exhaustive exercise-induced fatigue via forced swimming, an animal model of FM-like symptoms [55]. In an exhaustive exercise-induced fatigue animal model, Xianchu et al. reported that GSPE-supplemented mice increased swim time and fatigue threshold (as shown by decreased lactic acid, lactic dehydrogenase, and creatine kinase levels in serum) by increasing serotonergic and noradrenergic neurotransmissions [55]. Authors also found serum and skeletal muscle contained increased SOD, catalase (CAT), and total antioxidative capability (T-AOC) with decreased MDA levels, TNF-α, and IL-1β levels in mice treated with exhaustive exercise [55]. In terms of improving mitochondrial function, GSPEs supplementation in animals increased succinate dehydrogenase (SDH) and Na^+^-K^+^-ATPase activities in the skeletal muscles, which have shown to carry out anti-fatigue effects [55].

In a double-blind, randomized, crossover trial comparing three doses of anthocyanidins and placebo, over the course of three months, anthocyanins were found to have small yet significant results with an 80 mg/day dose on patients diagnosed with moderate to severe FM [51]. Patients showed significant decreases in sleep disturbances and fatigue levels from the baseline month to the last month of treatment [56]. The recommended daily dose of anthocyanins increased from 40 mg/day to 80 mg/day due to the optimal benefits seen at this dosage level [56]. While the results were encouraging, further research is warranted due to the small trial number (*n* = 12) involved [56]. Potentially higher doses of anthocyanins could also be further explored, making anthocyanin a drug rather than just a food supplement.

## 6. N-3 Polyunsaturated Fatty Acids

N-3 Polyunsaturated fatty acids (PUFAs) are integral components of phospholipids, the main building block of cell membranes. n-3 PUFAs consist of α-linolenic acid (ALA), eicosapentaenoic acid (EPA), and docosahexaenoic acid (DHA). DHA specifically is the most abundant component in neuronal membrane phospholipids [57]. Table 5 lists the effects of n-3 PUFAs on FM-like symptoms in animal and human studies.

In a thermally induced pain sensitivity mouse model, Veigas et al. reported that supplementation of concentrated fish oil (CFO) into the diet decreased central amplification and nociceptive sensitivity to heat-induced pain in the plantar paw region [58]. Such CFO’s analgesic properties may be, in part, mediated by suppression of the protein expression of c-fos (a marker of neuronal activation) and mRNA expression of ASIC1a, ASIC13, and TRPV1 [59]. In a double-blind placebo-controlled design, Fontani’s team investigated how EPA and DHA co-treatment over the course of 35 days could affect physiological/psychophysical parameters and clinical FM-like symptoms in female patients with musculoskeletal pain and FM [60]. Authors reported that compared to the placebo group, the n-3 PUFAs group had significant decreases in the AA/EPA ratio in blood and the number/pain intensity of painful tender points [60], while there were no changes in mood and sleep disturbance in patients compared to the placebo group. Although promising results were obtained, further research is necessary for subsequent trials with larger sample sizes, various dosages, and prolonged omega-3 PUFA treatment [60].

## 7. Naringin

Naringin and its neuroactive metabolite, naringenin, are two flavonoids seen in grapefruit and other citrus fruits [59,61]. Naringenin has been shown to cross the blood–brain barrier [59] and is well observed in human serum after ingestion due to its good bioavailability [60]. Much of the data features naringin’s neuroprotective, antioxidant, and anti-inflammatory effects, seen in various animal models of FM (Table 6). Table 6 list the effects of naringin and its bioactive compounds on FM-like symptoms in animals.

Anti-inflammatory mediated analgesic effects of naringenin were also seen in various hyperalgesic and stress-induced animal models. For example, in a forced swim-induced FM-like symptom mouse model, Ben-Azu et al. reported that repeated naringin administration to mice improved neurobehavioral activities, as shown by increased local activities, decreased immobility, decreased depression-like and anxiety-like behaviors, and increased % social preference and cognitive performance in animals when compared to controls [62]. Authors also reported naringin treatment resulted in (i) a decrease in acetylcholinesterase (AChE) activity, (ii) an increase in SOD, GPX, and CAT activities in the brain, and (iii) a decrease in MDA and nitrite levels in the brain [61]. These findings suggest that naringin treatment might be useful in producing functional behavioral effects through mechanisms related to the enhancement of cholinergic transmission, the antioxidant defense system, and the inhibition of lipid peroxidation and nitrosative processes.

Pinho-Ribeiros’ group demonstrated the anti-nociceptive effect of naringin in a variety of FM-like pain symptoms models that were induced by acetic acid, phenyl-p-benzoquinone (PBQ), formalin, complete Freud’s adjuvant (CFA), capsaicin, carrageenan, and PGE_2_ [60,63]. Intriguingly, naringenin administration significantly reduced inflammatory pain in animals via upregulating NO production with subsequent cyclic GMP-PKG-ATP-sensitive K^+^ channel signaling pathway [60]. Naringenin administration was also shown to prevent glutathione (GSH) reduction and inhibit hyperalgesic cytokines production (i.e., IL-33, TNF-α, IL-1β) as well as NF-κB activation in animals [60]. In a thermal (hot plate)-induced FM-like pain model, Xue et al. further confirm the anti-nociceptive and anti-inflammatory efficacy of naringenin in different FM-like symptom animal models (Table 6) [64]. Such anti-inflammatory effects of naringenin are verified by reduced proinflammation production (TNF-α, IL-1β, and IL-6) in the skin and leukocyte infiltration of paw edema in animals. In an exercise-induced fatigue-induced model, Zamanian et al. demonstrated that compared with the control group, naringin groups significantly increased exhaustive swimming time, increased blood glucose levels, as well as decreased LDH and MMP-9 levels, suggesting naringin’s anti-fatigue effects which may be attributed to its property in improving energy metabolism and reducing strenuous exercise-induced skeletal muscle damage [65,66].

## 8. Genistein

Soy phytoestrogen isoflavones are rich in soy products and have been found to have strong estrogenic properties and bind well to estrogen receptors (ER) [41]. Genistein and daidzein, bioactive phytoestrogen isoflavone in soybeans, preferentially binds to ERβ receptors expressed in neuronal and immune cells [44]. The analgesic, neuroprotective, immunomodulating, antioxidant, and anti-inflammatory effects have been well reported in animal studies [44]. Table 7 lists the effects of genistein on FM-like symptoms in animals. The release of substance P from nociceptive nerve fibers and activation of its receptor neurokinin 1 are important effectors in the transmission of pain signals. In an acid injection-induced FM-like animal model, Lin et al. showed that genistein administration led to antinociception for acid pain in muscle nociceptors of animals, via suppressing substance P-mediated inhibition of acid-sensing ion channel 3-selective current and phosphotyrosine kinase (PTK) activity [45,67]. Jie et al. demonstrated that compared with vehicle control, genistein treatment significantly reduced glutamate-evoked mechanical hypernociception in masseter muscles of animals, via suppression of the *N*-methyl-d-aspartate receptor of the NR2B subunit (pNR2B) and phosphorylated mitogen-activated protein kinase (pERK1/2) signaling pathways in the hippocampus [68].

## 9. Possible Molecular Mechanisms

**Figure 1:**
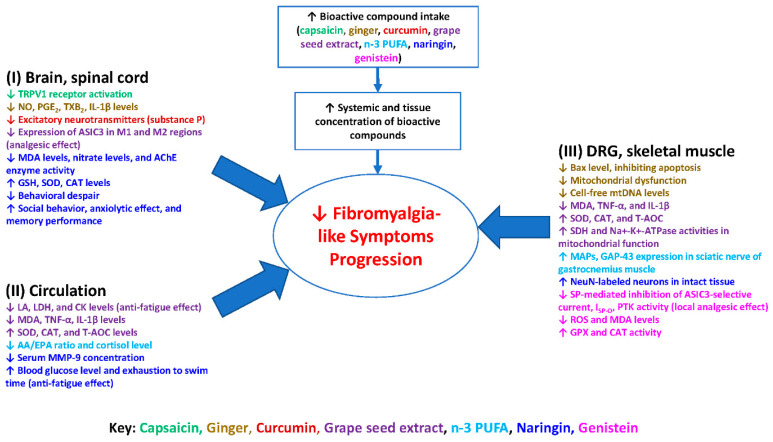
Diagram illustrates the potential actions of the bioactive compounds capsaicin, ginger, curcumin, grape seed extract, n-3 PUFA, naringin, and genistein on fibromyalgia-like symptoms. These compounds not only result in the production of pro-inflammatory cytokines, lipid peroxidation, and oxidative stress, but also result in upregulating antioxidant enzymes capacities in the nervous system, skeletal muscles, and circulatory system. The consequence is mitigation of the progression of fibromyalgia-like symptoms. Abbreviations: AA/EPA, arachidonic acid/eicosapentaenoic acid; CAT, catalase; CK, creatine kinase; CNS, central nervous system; DRG, dorsal root ganglion; GSH, glutathione; IL-1β, interleukin-1β; LA, lactic acid; LDH, lactate dehydrogenase; MAPs, muscle action potentials; MDA, malondialdehyde; MMP-9, matrix metalloproteinase-9; NO, nitric oxide; n-3 PUFA, n-3 polyunsaturated fatty acids; PGE2, prostaglandin E2; PNS, peripheral nervous system; ROS, reactive oxygen species; SDH, succinate dehydrogenase; SOD, superoxide dismutase; T-AOC, total antioxidant capacity; TNF-α, tumor necrosis factor-α; TXB2, thromboxane B2.

Figure 1 summarizes the possible molecular mechanisms of bioactive compounds in the nervous system (i.e., brain, spinal cord, dorsal root ganglia), skeletal muscles, and circulatory system (i.e., blood, plasma, or serum). Scientific evidence suggests these bioactive compounds may provide long-lasting therapeutic benefits that mitigate the progression of FM. The anti-nociceptive effects of these compounds appear to be mediated via the desensitization of specific membrane receptors, downregulation of inflammatory cytokines, and inhibition of antioxidant or anti-inflammation pathways. These bioactive compounds may treat FM-like symptoms by increasing free radical scavenging/antioxidant capabilities and enhancing neuronal survival, as well as decreasing ROS, neuroinflammation, and peripheral pro-inflammatory cytokine production. The overarching goal in modulating these aforementioned pathways is to bring balance to an imbalanced processing of central and peripheral pain in FM patients. These bioactive compounds have great potential as nonnarcotic anti-algesic treatment options for FM-like symptoms. Grape seed extract, n-3 PUFA, naringin, and genistein have been found to work similarly to current anticonvulsants, producing even more desirable effects by not only decreasing excitatory neurotransmitters but also providing neuroprotective and anti-inflammatory effects to nerves.

## 10. Summary and Future Direction

The results from the in vitro and animal studies included in this review demonstrate the numerous benefits of these food-derived bioactive components in treating FM-like symptoms; furthermore, a small number of human studies have been conducted to evaluate their therapeutic potential in patients with FM-like symptoms.

Animal studies have shown that these regularly consumed bioactive compounds have an effect on FM-like symptoms, as evidenced by decreased pain hypersensitivity and fatigue, as well as improved social behaviors. Further studies are warranted to allow meaningful comparison and quantification of the efficacy of these bioactive compounds on FM-like symptoms across studies, in terms of actual changes in antioxidant capacity, pain hypersensitivity, fatigue, and social behaviors.

It is worthy to note that stress has been shown to exacerbate symptoms in patients with FM. Fischer et al. reported that (i) increases in stress levels preceded increases in pain levels, and (ii) cortisol levels were positively correlated with pain levels in FM patients, suggesting cortisol may be involved in the diurnal fluctuation of pain levels in FM patients [69]. As of now, however, changes in cortisol levels and pain experienced in FM patients due to bioactive compounds are still unknown. Future research on how bioactive compounds may affect stress and cortisol in FM patients is warranted to address this knowledge gap.

Limitations of this review include a lack of robustness in the selected studies (and evidence) and the lack of human studies to corroborate or confirm the findings in animal studies on how these bioactive compounds would improve FM-like symptoms in patients. More rigorous animal and human studies are needed, as well as a quantitative report of the magnitude of changes in FM-like symptoms due to bioactive compounds across studies to highlight the strength of bioactive compounds in FM management.

Further work is required in developing and utilizing an ideal animal model of FM, particularly one that mimics key symptoms, comorbidities, absence of peripheral tissue pathology, and the predominance in females, which would enable researchers to study the effects of these bioactive compounds in a more accurate and specific manner. Further research that focuses on more comprehensive preclinical studies is necessary to fully elucidate the bioactive compounds’ multiple mechanisms of action, efficacy, safety, targeting, and bioavailability. The therapeutic value of bioactive compounds for FM patients in clinical studies or trials is an important but understudied area. Future lines of research should focus on how these bioactive compounds affect patients with FM and their various symptoms, including widespread pain, fatigue, sleep disruption, cognitive dysfunctions, and mood issues. The pattern of effects may guide therapeutic use but also shine light onto modes of action. It will also be important to study how these bioactive compounds might interact with currently used FM drugs, and if they can mitigate their side effects and associated long-term toxicity in FM patients. Such a nutritional approach would allow more inclusive treatment regimens to be an option for people with chronic FM-like symptoms, or for those facing other healthcare barriers such as prohibitive prescription drug costs.

## Figures and Tables

**Table 2:** Effects of ginger on fibromyalgia-like symptoms.

First Author, Year [ref]	Experimental Design and Treatments	Results
*In vitro study*		
Ha, 2012 [36]	BV-2 cells were cultured and activated with LPS (1 mg/mL) for 12 h. LPS was removed from the cells and indicated 6-shogaol (1, 5, 10 μM) was added. NMMA, an inhibitor of iNOS enzyme activity, was used as a positive control	Compared to control group, 6-shogaol groups:
		↓ iNOS expression and release of NO
		↓ microglial activation
Hosseinzadeh, 2017 [37]	C28I2 human chondrocytes pretreated with GE (5, 25 μg/mL) for 24 h, followed by incubation with IL-1β (10 ng/mL) for 24 h	Compared to vehicle group, GE groups:
		↑ anti-oxidant enzyme gene expression
		↓ reactive oxygen species
		↓ lipid peroxidation
		↓ Bax/Bcl ratio, inhibiting apoptosis
		↓ caspase-3 activity
*Animal studies*		
Montserrat-de la Paz, 2018 [38]	Intermittent cold stress (ICS)-induced FM model	Compared to control group, GR groups:
	Female C57BL/6 J mice (*n* = 48, 5-week-old)	↓ mechanical and thermal allodynia
		↓ mechanical hyperalgesia
	Treatments: healthy control, ICS group, ICS + APAP (40 mg/kg/day), ICS + GR (0.5%, 1%), and ICS + GR (0.5%) + APAP (40 mg/kg/day) supplemented with standard diet for 8 weeks	↑ improved behavioral changes related to cognitive disturbances associated with pain, anxiety, and depression ↓ proinflammatory mediators such as NO, PGE2, TXB2, IL-1β in LPS-stimulated macrophages
		↑ synergism with APAP leading to further reduction in FM-like symptoms
Fajrin, 2019 [39]	Complete Freud’s Adjuvant (CFA)-induced FM-like model	Compared to control group, RGO groups:
	Male mice (*n* = 48, 8-week-old)	↓ hyperalgesia in a dose-dependent manner
	Treatments: sham, negative control, RGO doses at 100, 200, 400 and 600 mg/kg orally 1x/day for 7 days	↑ thermal response threshold
Abolaji, 2017 [40]	Chlorpyrifos (CPF)-induced FM-like model	Compared to the control group, 6-GRF groups:
	Female Wistar rats (*n* = 70, 100–125 g)	↓ H2O2 and MDA levels
		↑ catalase, SOD, GPX activity
	Treatments: control with corn oil only (2 mL/kg BW), 6-GRF (100 mg/kg BW), CPF dissolved in corn oil (5 mg/kg BW), CPF (5 mg/kg BW) and 6-GRF (50 mg/kg BW) concomitantly, CPF (5 mg/kg BW) and 6-GRF (100 mg/kg BW) concomitantly 1x/day for 35 days after CPF toxicity	↓ NO, MPO, TNF-α
		↓ caspase-3
Zhang, 2016 [41]	DSS-induced chronic colitis model	Compared to control group, GDNP group:
	Female FVB/NJ mice (*n* = 7, 6–8-week-old)	↑ healing in wounded intestinal epithelial monolayers
		↑ proliferation of IECs
	Groups including control with normal feeding and GDNPs 2 (300 μL of 1 mg/mL solution) by oral gavage 1x/day for 18 weeks	↓ TNF-α, IL-6, IL-1β, MPO
		↑ IL-10, IL-22

Abbreviations: APAP, paracetamol; BW, birth weight; CFA, complete Freud’s Adjuvant; CPF, chlorpyrifos; DSS, dextran sodium sulfate; FM, fibromyalgia; ICS, intermittent cold stress; IL-1β, interleukin-1β; GE, ginger extract; GPX, glutathione peroxidase, GR, ginger rhizome; GRF, gingerol-rich fraction; H_2_O_2_, hydrogen peroxide; iNOS, inducible nitric oxide synthase; LPS, lipopolysaccharide; MDA, malondialdehyde; MPO, myeloperoxidase; NO, nitric oxide; PGE_2_, prostaglandin E_2_; RGO, red ginger oil; SOD, superoxide dismutase; TNF-α, tumor necrosis factor-α; TXB_2_, thromboxane B2; ↑ increase; ↓ decrease; ↔ no difference.

**Table 3:** Effects of curcumin on fibromyalgia-like symptom in animal studies.

First Author, Year [ref]	Experimental Design and Treatments	Results
*Animal studies*		
Yang, 2017 [43]	Dextran sodium sulfate (DSS)-induced FM-like model	Compared to naive control group, curcumin group:
	Male SD rats (*n* = 57, 190–210 g)	↓ visceral hyperalgesia in dose-dependent manner (no significant effect with lower curcumin dose)
	Treatments: naive control, DSS + saline, and DSS + curcumin (20, 60 mg/kg) by oral gavage 1x/day for 10 days, beginning 3 days after initiation of DSS	↓ TRPV1 expression in DRG neurons
		↓ TRPV1 protein level in inflamed colon
Zhi, 2013 [44]	Colorectal distension (CRD)-induced FM-like (VMR) model	Compared to vehicle group, curcumin group:
		↓ CRD-induced VMRs indicating a decrease in GI nociception
	Adult male SD rats (*n* = 4, 250–300 g)	↓ visceral hyperalgesia
	Treatments: control, vehicle, and curcumin (4 mg/kg⋅min) infusion for 3 min	↓ TRPV1 activation in primary afferent neurons in concentration-dependent manner
	Jejunal afferent firing in ex vivo jejunum preparations model	Compared to control group, curcumin groups:
	Adult male Kunming mice (*n* = 25, 20–30 g)	↓ TNBS-induced hypersensitivity of jejunal afferents
	Treatments: control, curcumin at 1, 3, 10, and 30 μmol/L given extra- and intra-luminally in naïve and TNBS-treated mouse jejunum	↓ capsaicin-induced rise in intracellular calcium and inward currents in mouse or rat DRG neurons
Fattori, 2015 [45]	KO_2_-induced superoxide anion-induced FM-like model	Compared to control group, curcumin groups:
		↓ mechanical and thermal hyperalgesia
	Male Swiss mice (25–30 g)	↓ superoxide anion-induced leukocyte recruitment in peritoneal cavity
		↓ MPO activity, oxidative stress, IL-1β and TNF-α production in paw skin
	Treatments: vehicle (2% DMSO in saline) i.pl., KO_2_ i.pl + saline s.c., KO_2_ i.pl. + curcumin (3, 10, or 30 mg/kg) s.c. 1 h before KO_2_ stimulus	↓ NF-κB activation in paw skin
		↑ IL-10 production, and HO-1 and Nrf2 mRNA expression in paw skin

Abbreviations: BW, body weight, CRD, colorectal distension; DMSO, dimethyl sulfoxide; DRG, dorsal root ganglia; DSS, dextran sodium sulfate; HO, heme oxygenase; IL-1β, interleukin-1β; i.pl., intraplantar; KO_2_, potassium superoxide; Nrf2, nuclear factor erythroid 2-related factor 2; s.c. subcutaneous; SD, Sprague-Dawley; TNBS, trinitrobenzene sulfonic acid; TNF-α, tumor necrosis factor-α; TRPV1, transient receptor potential vanilloid-1; VMR, viscero-motor response; ↑ increase; ↓ decrease; ↔ no difference.

**Table 4:** Effects of grape seed extract on fibromyalgia-like symptoms.

First Author, Year [ref]	Experimental Design and Treatments	Results
*In vitro studies*		
Fujishita, 2009 [47]	Oxidative stress-induced hippocampal neuronal cell death model	Compared to vehicle group, the GSE groups:
	Neurons treated with GSE (0–100 µg/mL) for 2, 6 and 12 h. Neurons pre-incubated for 24 h with GSE-treated ACM in presence or absence of anti-IL-6 antibodies (50 ng/mL). Then, neurons were stimulated with H_2_O_2_ in presence or absence of IL-6 with and without anti-IL-6 antibody for 2 h	↑ mRNA expression of IL-6, COX-2, IL-1α,
		in astrocytes with oxidative stress status
		Protected against neuronal death induced by
		oxidative stress
Narita, 2011 [48]	Hippocampal neurons at 8 DIV treated with 50 µM glutamate for 30 min, in presence or absence of 0.01, 0.1, 1.0, or 10 ng/mL of GSE	Compared to vehicle group, the GSE groups:
		Protected Erk1/2 phosphorylation
		Protected dendritic arborization and augmented cell survival
		↓ caspase-3 activity
*Animal studies*		
Mun, 2010 [42]	i.p. injection in gastrocnemius muscle of acidic saline-induced FM-like model	Compared to control group, proanthrocyanidin group:
	(*n* = 15)	
	Female male SD rats (*n* = 15, 250–320 g)	↑ anti-hyperalgesic effect in injected paw and contralateral paw
	Treatments: control, acidic saline i.p. injection, oligomeric proanthrocyanidin complexes at 300 mg/kg injection i.p. on day 7	↓ expression of acid sensing ion channel 3 in brain M1 and motor cortex area
Xianchu, 2018 [49]	Exhaustive exercise-induced FM model	Compared to control group, GSPE-M and GSPE-H groups,
		↓ fatigue by prolonging the time to exhaustion in forced swimming test
		↓ lactic acid, LDH, and CK in serum
	Male ICR mice (*n* = 32, 8-week-old, 25 g)	↓ MDA, TNF-α, and IL-1β in serum and skeletal
		muscle of mice
		↑ SOD, CAT, and T-AOC in serum and skeletal
	Treatments: control, low-dose GSPE-L group (1 mg/kg/day), medium-dose GSPE-M group (50 mg/kg/day), and high-dose GSPE-H group (100 mg/kg/day) for 28 days	muscle of mice
		↑ SDH and Na+-K+-ATPase activities in
		mitochondrial function of skeletal muscle
*Human study*		
Edwards, 2000 [50]	Double-blind, randomized, crossover trial with moderate to severe primary FM (*n* = 12)	Compared to the placebo group, anthocyanidins at 80 mg daily group:
	Treatments: placebo, 40 mg, 80 mg, and 120 mg anthocyanidins daily. Each dose for 3 months	↓ fatigue and sleep disturbance in 80 mg dose group

Abbreviations: CAT, catalase; CK, creatinine phosphokinase; COX2-, cyclooxygenase-2; FM, fibromyalgia; GSE, grape seed extract; GSPE, grape seed proanthrocyanidin extract; IL-1β, interleukin-1β; i.p., intraperitoneal; LDH, lactate dehydrogenase; MDA, malondialdehyde; SD, Sprague-Dawley; SDH, succinate dehydrogenase; SOD, superoxide dismutase; T-AOC, total antioxidative capability; TNF-α, tumor necrosis factor-α; ↑ increase; ↓ decrease; ↔ no difference.

**Table 5:** Effects of omega-3 PUFAs on fibromyalgia-like symptoms.

First Author, Year [ref]	Experiment Design and Treatment	Results
*Animal * *studies*		
Veigas, 2011 [53]	Heat-induced FM-like model	Compared to SO group, CFO groups
	C57BL/6J male mice (*n* = 60)	↓ sensitivity to heat induced pain in plantar paw region
	Treatments: 4% regular fish oil diet (FO), 4% concentrated fish oil diet (CFO), 5% safflower oil diet (SO) ad libitum for 6 months	↓ c-fos protein immunoreactivity and mRNA expression of ASIC1a, ASIC13, and TRPV1 in DRGs
*Human study*		
Fontani, 2010 [54]	Double-blind placebo-controlled design	Compared to placebo group, n-3 PUFA group:
		↓ pain intensity
		↓ number of positive tender points
	Treatments: placebo group (*n* = 23): 4 g of oleic sunflower oil daily for 35 days and n-3 PUFA group (*n* = 23): 4 g oil (2.8 g of omega-3 polyunsaturated fatty acids, EPA +DHA in a 2:1 ratio; 1,60 g EPA, 0.8 g DHA, 0.4 g of other types of omega-3 polyunsaturated fatty acids: alpha linolenic, stearidonic, eicosatetraenoic and ocosapentaenoic acid) daily for 35 days	↓ pain intensity
		↔ superficial thermal (heat and cold)
		↓ anger-hostility and depression↓ in AA/EPA, AA, cortisol↑ EPA, pain area (%)

Abbreviations: ASIC, acid-sensing ion channel; CFO, concentrated fish oil; DHA, docosahexaenoic; DRG, dorsal root ganglion; EPA, eicosapentaenoic acid; FO, fish oil; PUFA, polyunsaturated fatty acid; SO, safflower oil; TRPV1, transient receptor potential cation channel subfamily V member 1, ↑ increase; ↓ decrease; ↔ no difference.

**Table 6:** Effects of naringin on fibromyalgia-like symptoms.

First Author, Year [ref]	Experimental Design and Treatments	Results
*Animal studies*		
Ben-Azu, 2019 [56]	Forced swim-induced FM-like model Male Swiss mice (*n* = 50) Treatments: vehicle (10 mL/kg BW, i.p.), naringin (2.5, 5 and 10 mg/kg BW, i.p.), diazepam (2 mg/kg BW, i.p.), donepezil (1 mg/kg BW, i.p.) and imipramine (15 mg/kg BW, i.p.) for 7 days	Compared to vehicle group, naringin groups:
		↑ locomotor activity
		↓ depressant and anxiety
		↑ % social preference
		↑ cognitive performance
		↓ AChE enzyme activity in brain
		↑ GSH, SOD, CAT levels in brain
		↓ MDA and nitrate levels in brain
Pinho-Ribeiro, 2016 [57]	Acetic acid- and PBQ-induced FM-like visceral pain model Male Swiss mice Treatments: vehicle, Nar (16.7–150 mg/kg p.o.) given 30 min before stimulus Formalin-induced FM-like pain model Male Swiss mice Treatments: vehicle, Nar (50 mg/kg, p.o.) given 30 min before stimulus Capsaicin- and CFA-induced overt FM-like pain models Male Swiss mice Treatments: vehicle, Nar (50 mg/kg, p.o.) given 30 min before nociceptive stimulus. Carrageenan-, CFA-, capsaicin-, and PGE_2_-induced mechanical hyperalgesia Male Swiss mice Treatments: vehicle, Nar p.o. given 30 min before stimulus	Compared to vehicle group, Nar groups:
		↑ antinociceptive effect induced by acid, formalin, and capsaicin- and CFA
		↓ mechanical hyperalgesia induced by carrageenan, capsaicin, CFA, and PGE2
		↑ NO/cGMP/PKG/ATP sensitive K^+^ channel signaling pathway
		↑ GSH production of plantar skin tissue
		↓ IL-33, TNF-α, IL-1β production, and NF-κB activation of paw skin tissues
Zamanian, 2016 [59]	Exercise-induced fatigue-induced FM model Female Wistar rats (*n* = 50) Treatments: control, vehicle, 40, 80, and 160 mg naringin/kg/day for 30 days	Compared to vehicle group, naringin groups:
		↑ exhaustion swimming time
		↓ LDH activity and serum MMP-9 levels
		↑ blood glucose levels
Xue, 2019 [60]	Hot plate-induced FM-like pain model	Compared to vehicle group, Nar groups:
	Acetic acid-, glutamate-, capsaicin-, capsaicin-, and formalin-induced FM-like pain models	↑ antinociceptive effect against thermal and chemical-induced pain
	Treatments: vehicles, Nar (25–75 mg/kg) p.o., before stimulus	↓ capsaicin-induced paw licking number
	Carrageenan-induced FM-like inflammatory model	↓ carrageenan-induced peritoneal leukocyte infiltration of paw edema
	Treatments: vehicles, Nar (25–75 mg/kg) p.o., given 30 min before stimulus	↓ TNF-α, IL-1β, and IL-6 levels of skin

Abbreviation: AChE, acetylcholine esterase; BW, body weight; CAT, catalase; CFA, complete Freud’s Adjuvant; FM, fibromyalgia; GSH, glutathione; IL-1β, interleukin-1β; IL-6, interleukin-6; i.p., intraperitoneal; i.pl., intraplantar; LDH, lactate dehydrogenase MDA, malondialdehyde; MMP-9, matrix metalloproteinases-9; NF-κB, nuclear factor kappa B; p.o., post oral; PBQ, phenyl-p-benzoquinone; PGE_2_, prostaglandin E_2_, TNF-α, tumor necrosis factor-α, NAR; naringenin; SOD, superoxide dismutase; ↑ increase; ↓ decrease; ↔ no difference.

**Table 7:** Effects of genistein on fibromyalgia-like symptoms.

First Author, Year [ref]	Experimental Design and Treatment	Results
*Animal studies*		
Lin, 2011 [61]	Acetic acid-induced FM-like pain model	Compared to vehicle group, genistein group:
	C57/BL6 mice (8–12-week-old)	
	Treatments: vehicle treated with substance P (SP to induce current), genistein (30 µM), SP + genistein, SP + daidzein (30 µM) for 10 min on DRG neurons	↓ SP-mediated inhibition of ASIC3-selective current and PTK activity of muscle dorsal root ganglion neuron
Jie, 2018 [68]	Glutamate-induced masseter muscle FM-like pain model	Compared to control group, genistein groups:
		↓ mechanical hypernociception at high genistein dose
	Female Sprague-Dawley OVX rats	Partially reversed E2-potentiated glutamate-evoked hypernociception of masseter muscle
	Treatments: no injections (control); E2 vehicle + genistein, E2 + genistein vehicle, and E2 + genistein (2–60 mg/kg) for 12 days.	↓ pNR2B and pERK1/2 expression in hippocampus

Abbreviation: ASIC3, acid-sensing ion channel 3; DRG, dorsal root ganglion; E2, 17β-estradiol; i.m., intramuscular; OVX, ovariectomized/ovariectomy; pERK1/2, phosphorylated mitogen-activated protein kinase; PTK, phosphotyrosine kinase; pNR2B, *N*-methyl-d-aspartate receptor of the NR2B subunit; SP, substance P; ↑ increase; ↓ decrease; ↔ no difference.
